# The Molecular Basis of Calcium and Phosphorus Inherited Metabolic Disorders

**DOI:** 10.3390/genes12050734

**Published:** 2021-05-13

**Authors:** Anna Papadopoulou, Evangelia Bountouvi, Fotini-Eleni Karachaliou

**Affiliations:** Third Department of Pediatrics, University General Hospital “ATTIKON”, Medical School, National and Kapodistrian University of Athens, 124 62 Athens, Greece; boudouvi@yahoo.gr (E.B.); fenkar1@hotmail.com (F.-E.K.)

**Keywords:** Calcium Sensing Receptor (CaSR), Parathyroid hormone (PTH), Vitamin D, Vitamin D Receptor (VDR), Fibroblast growth factor 23 (FGF23), calcium metabolism, phosphorus metabolism, rare diseases

## Abstract

Calcium (Ca) and Phosphorus (P) hold a leading part in many skeletal and extra-skeletal biological processes. Their tight normal range in serum mirrors their critical role in human well-being. The signalling “voyage” starts at Calcium Sensing Receptor (CaSR) localized on the surface of the parathyroid glands, which captures the “oscillations” of extracellular ionized Ca and transfers the signal downstream. Parathyroid hormone (PTH), Vitamin D, Fibroblast Growth Factor (FGF23) and other receptors or ion-transporters, work synergistically and establish a highly regulated signalling circuit between the bone, kidneys, and intestine to ensure the maintenance of Ca and P homeostasis. Any deviation from this well-orchestrated scheme may result in mild or severe pathologies expressed by biochemical and/or clinical features. Inherited disorders of Ca and P metabolism are rare. However, delayed diagnosis or misdiagnosis may cost patient’s quality of life or even life expectancy. Unravelling the thread of the molecular pathways involving Ca and P signaling, we can better understand the link between genetic alterations and biochemical and/or clinical phenotypes and help in diagnosis and early therapeutic intervention.

## 1. Introduction

The investigation of the genetic basis of Calcium (Ca) and Phosphorus (P) metabolic inherited disorders brings to light, piece by piece, precious information regarding the complexity and function of a huge molecular circuit, in which these minerals play a leading or supporting role, depending on the cellular demands. Although being recognized for their involvement in skeletal formation and maintenance, Ca and P participate in many biological processes. Their critical role for human health is mirrored by their serum near-constant concentrations, achieved by a well-orchestrated homeostatic system, which comprises the parathyroid glands, the intestine, the kidneys and the skeleton. To date, inherited metabolic disorders of Ca/P metabolism are mainly rare, presenting with a wide range of clinical manifestations, some of latter being common among diseases of different etiologies. Therefore, they require different therapeutic approaches. In this review, we provide an overview of the Ca/P signalling pathway and discuss the genetic basis of known inherited diseases associated with extracellular Ca sensing mechanisms, Parathyroid hormone (PTH) production and action, Vitamin D metabolism and Fibroblast Growth Factor 23 (FGF23) synthesis and function.

## 2. Calcium and Its Partners

Calcium (Ca) is the most abundant mineral in human bone tissue and critically important to human health [1]. It participates in bone formation and maintenance, muscle contraction, neuron depolarization, blood coagulation and intracellular processes, including signal transduction, production and secretion of hormones. Almost 1000 mg/24 h of Ca enter the body of an average adult, depending on the quantity of milk and other dairy products consumed. The recommended Ca intake for infants is 200 mg/24 h which gradually increases in childhood, while the highest requirements are noted during adolescence and individuals over 50 years old [2]. About 40% of dietary Ca is absorbed from the gastrointestinal tract via a paracellular pathway and an active transcellular pathway both potentially regulated by 1,25(OH)_2_D_3_ [3,4,5]. Half of the absorbed Ca leaves the body in urine and feces. There are 3 major pools of Ca in the body: (a) Within cells, (b) blood and extracellular fluid and (c) bones, where the vast majority of body Ca is stored.

Within bones, 99% of Ca is found in the mineral phase as hydroxyapatite [Ca_10_(PO4)_6_(OH)_2_], but the remaining 1% is in a pool that can rapidly exchange with extracellular Ca to ensure the Ca homeostasis. The skeleton turns over about 250 mg/day of Ca, while approximately 10,000 mg/day is filtered at the glomerulus and most is reabsorbed by the renal tubules [6]. Approximately half of Ca in blood and extracellular fluid is bound to proteins (~45% albumin) or complexed with ions (~10%), including phosphates and ~45% circulates as a free, ionized fraction (Ca^2+^). The biologic effect of Ca is determined by Ca^2+^ rather than the total Ca. The serum level of Ca is closely regulated with a normal total Ca of 2.2–2.7 mmol/L (8.8–10.8 mg/dL) and a normal Ca^2+^ of 1.12–1.23 mmol/L (4.8–4.92 mg/dL). The concentration of Ca^2+^ in blood and extracellular fluid is normally almost invariant at approximately 1 mM, or 1000 to 10,000 times the basal concentration of Ca^2+^ within cells. Deviations above or below the normal range, frequently, lead to serious diseases. Contrary to the tight serum normal range, intracellular Ca^2+^ concentrations fluctuate greatly, from roughly 100 nM to greater than 1 μM, due to release from cellular stores or influx from extracellular fluid. These fluctuations assign Ca direct or indirect responses to both cellular needs and serum Ca homeostasis [1].

Maintaining constant Ca^2+^ concentrations in blood requires the collaboration of primary regulating hormones, PTH and 1,25(OH)_2_D_3_, receptors and enzymes involved in Ca metabolic pathway. Being the first player of this cascade, the Calcium Sensing Receptor (CaSR) captures the oscillations of serum Ca^2+^ and, along with the activity of the parathyroid glands, regulate PTH synthesis and secretion. Next, PTH acts on the target organs, mainly on the kidneys and bones through its receptor and indirectly on the intestine via regulation of 1,25(OH)_2_D_3_. Defects of the molecular components of this circuit are frequently the cause of genetic inherited disorders.

### 2.1. Calcium Sensing Receptor

**Figure 1:**
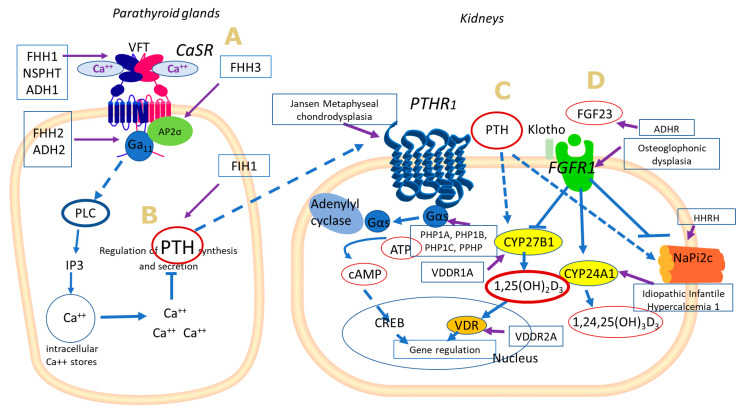
Schematic illustration of selective molecular alterations associated with inherited disorders of Ca/P metabolism. (**A**) In the parathyroid gland, CaSR activation by Ca^2+^ or other compatible ligands, induces Gα_q/11_-mediated activation of phospholipase C (PLC), increases inositol 1,4,5-trisphospate (IP3) and leads to intracellular Ca^2+^ mobilization, which acts as a secondary messenger of different cell-dependent actions, such as PTH synthesis regulation. (**B**) PTH acts through its receptor PTHR1 at target organs (kidney), and activates via G proteins, the axis Gα_S_–adenylyl cyclase–cAMP–protein kinase A (PKA). The produced cAMP acts as cellular second messenger promoting the transcription of target genes. (**C**) PTH promotes 1,25(OH)_2_D_3_ synthesis via CYP27B1 activation and inhibits Pi reabsorption by inhibiting NaPi-2c (and NaPi-2a) cotransporter. (**D**) FGF23 mediates its actions through its receptor FGFR1 and inhibits 1,25(OH)_2_D_3_ renal synthesis and Pi reabsorption while it promotes 1,25(OH)_2_D_3_ catabolism via activation of CYP24A1. Genetic defects associated with alterations of the proteins illustrated in this scheme are marked in the respective boxes.

CaSR comprises a fundamental contributor to extracellular Ca^2+^ homeostasis, modulating the synthesis/secretion of PTH in an inverse manner, reducing Ca^2+^ renal reabsorption and interfering in bone development and lactation [7]. It binds to various physiological ligands and is expressed in many calcitropic and non-calcitropic tissues, yet more abundantly in cells of the parathyroid glands and the renal tubular cells. The receptor is a member of family C G-protein coupled receptors (GPCRs) characterized by a large extracellular N-terminal domain responsible for ligand binding and receptor dimerization, seven transmembrane segments and an intracellular C-terminal domain that couples to G proteins and other signal transduction molecules [8]. Understanding how Ca and other extracellular cations interact with the extracellular portion of the CaSR is not well-understood. It has been proposed that the binding of Ca^2+^ or other compatible ligands at the cleft between the Venus Flytrap (VFT) lobes in the extracellular domain induces rotation along the dimer interface (Figure 1) [9,10]. VFT consists of a large, nutrient-binding bilobed domain, located in the N-terminus part of the protein, adjacent to the C-side of signal peptide [11]. Such a conformational change could, thus, alter the spatial configuration of the membrane-spanning portion of the receptor and modulate signal transduction [8,11,12].

In the parathyroid gland, CaSR activation induces Gα_q/11_-mediated activation of phospholipase C (PLC), increases inositol 1,4,5-trisphospate (IP3) and leads to intracellular Ca^2+^ mobilization, which acts as a secondary messenger of different cell-dependent actions [11]. Cytoplasmic Ca^2+^ inhibits cyclic AMP (cAMP) production both directly by downregulating adenylyl cyclase (AC) and indirectly via prostaglandins, leading to suppression of PTH synthesis from the parathyroid chief cells. In conditions of blood Ca^2+^ depletion, the CaSR remains inactivated and no suppression of PTH occurs, thus promoting PTH synthesis and secretion [13]. In the kidneys, CaSR activation leads to a decrease in Ca^2+^ reabsorption.

Very small changes from baseline in blood Ca^2+^ concentration in persons with normal renal and parathyroid gland function elicit large changes in plasma PTH levels. The response occurs within seconds to minutes and provides a robust mechanism for rapidly maintaining constant blood Ca^2+^ levels. It should be noted that modulation of signal transduction through CaSR may also be influenced by interactions of CaSR with scaffolding proteins or by the heterogeneity among parathyroid cells with respect to the suppressive effects of Ca^2+^ on PTH release [9]. For instance, changes in caveolin-1 and filamin expression, two scaffolding proteins that interact with CaSR, may blunt the effect of Ca^2+^ on PTH synthesis or even affect cell cycle proliferation in parathyroid tissue [14,15]. Furthermore, heterogeneity among parathyroid cells could account for the variations in the response of patients with primary or secondary hyperparathyroidism to standardized intravenous infusion of calcium [16].

### 2.2. Parathyroid Hormone

Parathyroid Hormone (PTH) is initially synthesized as a prepropeptide which, after 2 proteolytic cleavages gives rise to the active 84-amino-acid peptide (intact PTH, iPTH) [17]. Several factors regulate its biosynthesis such as Ca^2+^, 1,25(OH)_2_D_3_ and Pi. The circulating half-life of the molecule is less than 5 min. Along with iPTH, a heterogeneous collection of peptides corresponding to the amino (N-), middle and carboxyl (C-) regions of the molecule are produced. The C-terminal PTH fragments, are present in the circulation in large amounts and being recognizable by specific receptors (CPTHRs) [18]. Normally, these fragments circulate in blood at concentrations higher than that of iPTH, and at much higher levels in renal failure. The normal levels of serum iPTH range between 10.0 to 60.0 pg/mL [19].

The principal role of PTH is to increase serum Ca^2+^ concentrations by acting upon its receptor which is highly expressed in bone and kidney. The classic PTH receptor is a member of the GPCR family. It is designated type 1 PTH/PTHrP receptor (PTHR1) as it recognizes the amino-terminus of PTH and the homologous terminus of the parathyroid hormone-related protein (PTHrP) with similar or indistinguishable affinity [18]. This receptor, via G proteins, can activate at least two signal transduction pathways: Gα_S_–adenylyl cyclase–cAMP–protein kinase A (PKA), where cAMP is the cellular second messenger, and Gαq–phospholipase C (PLC) β–IP3–cytoplasmic Ca^2+^–protein kinase C pathway protein kinase C (PKC). The direction of the regulation appears to depend on cell type and PTH concentration [20].

In the kidney, PTH enhances reabsorption of Ca^2+^, predominantly in the distal tubule and the thick ascending limb resulting in minimal losses of Ca^2+^ in urine. Furthermore, in the renal proximal tubule PTH inhibits the reabsorption of P [19]. In bone, PTH, via its receptor expressed on osteoblasts and stromal cells, has been considered to have either a catabolic or anabolic effect [21]. The anabolic effect is exerted by low and intermittent concentrations of PTH, and has been applicable to osteoporosis treatment by daily injections of PTH. On the other hand, high levels of PTH, as in primary and secondary hyperparathyroidism, lead to bone resorption. Finally, PTH promotes intestinal Ca absorption indirectly by up regulating renal 25-vitamin D 1α-hydroxylase, the enzyme responsible for converting 25(OH)D in its active form.

### 2.3. Vitamin D

Vitamin D is one of the most important orchestrates of Ca homeostasis. It is a fat-soluble secosteroid formed mostly in human skin by exposure to sunlight, while a small percentage comes directly from diet [22,23]. Vitamin D3 is produced photochemically in the skin from the precursor molecule 7-dihydroxycholesterol (7-DHC). Vitamin D2 (ergocalciferol), the exogenous form of vitamin D, is produced by ultraviolet irradiation of the plant sterol ergosterol and is acquired through the diet. The amount of Vitamin D recommended by the US Institute of Medicine, is 400–800 IU/day, depending on the ethnicity, the age and the socioeconomic status of the population [24,25]. Furthermore, under physiological particular conditions, such as pregnancy and lactation, vitamin D doses are adjusted [24,26,27].

**Figure 2:**
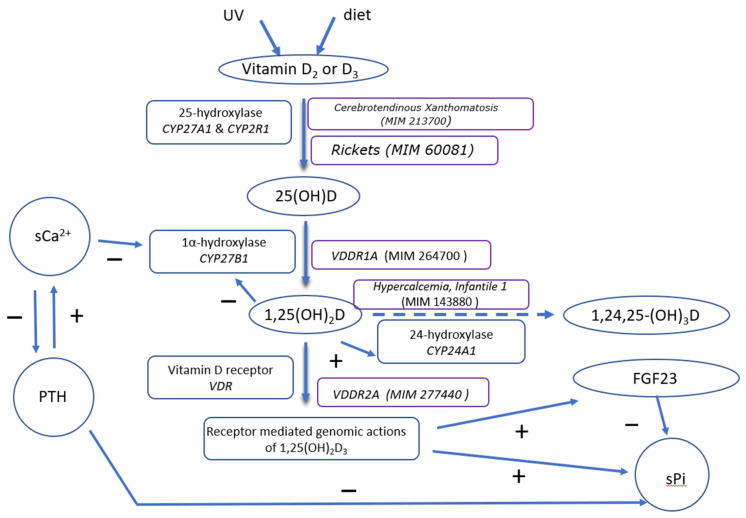
Schematic illustration of Vitamin D metabolism. Vitamin D resulting either from cutaneous synthesis or, directly from the diet, is metabolized to 25(OH)D by the liver mitochondrial 25-hydroxylase (CYP27A1), and the microsomal 25-hydroxylase (CYP2R1). 25(OH)D is further metabolized to 1,25(OH)_2_D, by the renal 1α-hydroxylase (CYP27B1). 1,25(OH)_2_D may exerts its biologic effects through its receptor (VDR) or be catabolized by the 24-hydroxylse (CYP24A1). The net effect of 1,25(OH)_2_D on Ca/P metabolism is the increase in Ca/P serum concentration. PTH on the other hand, ensures increased levels of sCa and decreased levels of sPi. In this figure, the genetic defects associated with alterations in the Vitamin D metabolic pathway are marked in boxes, next to the respective enzymes.

Both vitamin D3 and vitamin D2, must be further metabolised to be active. Their first hydroxylation at the carbon in position 25 produces 25(OH)D or calcidiol [28] (Figure 2). This conversion takes place in the liver by cytochrome P450 proteins, the mitochondrial 25-hydroxylase, CYP27A1, and the microsomal 25-hydroxylase, CYP2R1. Mutations in the genes encoding for the conversion enzymes are associated with rare inherited diseases such as cerebrotendinous xanthomatosis (MIM 213700) [29] (gene *CYP27A1*) without a defected vitamin D metabolism-and Vitamin D Hydroxylation Deficient Rickets type 1B (VDDR1B; MIM 60081) (gene *CYP2R1*) [30].

While, 25(OH)D is the main circulating metabolite of vitamin D with a half-life of several weeks and its concentration in serum reflects the vitamin D stores in man. Appropriate vitamin D levels are essential for proper bone health in early life and reducing the risk of developing osteoporosis later. The demarcation line between sufficient and insufficient vitamin D status is not very clearly defined. The Endocrine Society, defines as deficient, vitamin D status 25(OH)D serum levels in adults less than 20 ng/mL (<50 nmol/L); while the UK Scientific Advisory Committee on Nutrition (SACN) less than 11 ng/mL (25 nmol/L) and other agencies less than 13 ng/mL (30 nmol/L) [31].

In the proximal tubule of the kidney 25(OH)D is further metabolized in 1,25 (OH)_2_D_3_ by 1α-hydroxylase (gene: *CYP27B*), yielding the biologically active form of vitamin D. Its half-life is a few hours. Several types of cells, including cells from the decidua and placenta, keratinocytes, activated macrophages, prostatic and colon epithelial cells, and vascular endothelial and smooth muscle cells are known to express 1α-hydroxylase and produce 1,25(OH)_2_D_3_ [32]. The synthesis of 1,25(OH)_2_D_3_ is tightly regulated; stimulated by PTH and inhibited by high concentrations of Ca, P, FGF23 and 1,25(OH)_2_D_3_ itself. The normal serum concentration of 1,25(OH)_2_D_3_ is ranged between 20–60 pg/mL. Mutations in *CYP27B* are associated with Vitamin D Hydroxylation Deficient Rickets, type 1A (VDDR1A) (MIM 264700) [33]. The phenotypic and laboratory features of this disorder are presented in detail below. Moreover, in the kidney, the enzyme 25-hydroxyvitamin D- 24-hydroxylase, CYP24A1 converts 1,25(OH)_2_D_3_ to 1,24,25-trihydroxyvitamin D in a series of catabolic steps that lead to the inactivation of 1,25(OH)_2_D_3_ [34,35]. The gene transcription is mostly regulated by 1,25(OH)2D and PTH while mutations in *CYP24A1* are associated with Idiopathic Infantile Hypercalcemia type 1 (MIM 143880) [36].

While, 1,25-dihydroxy vitamin D, is transported in blood, bound to specific vitamin D-binding protein and, to a lesser amount, to albumin. Vitamin D mediates its biological effects through its own member of the nuclear hormone receptor superfamily, the vitamin D receptor (VDR) [37,38]. VDR is expressed in most organs including the intestine, bone, kidney, and parathyroid gland cells, as well as in brain, heart, skin, gonads, prostate and the breast. It is a transcription factor, and a member of the ligand-activated receptor superfamily that plays a major role in Ca metabolism. The receptor binds many vitamin D metabolites, and 1,25(OH)_2_D_3_ has the highest affinity. Upon activation by 1,25(OH)_2_D_3_, the VDR complexes with other nuclear hormone receptors and binds to specific DNA sequences called vitamin D response elements (VDRE) in the promoter of hormone-responsive genes, such as osteocalcin, p21, 24-hydroxylase, PTH [39]. It is estimated that VDR activation may regulate directly and/or indirectly the expression of a very large number of genes (0.5–5% of the total human genome, i.e., 100–1250 genes) [40].

The principal effect of 1,25(OH)_2_D_3_ is the stimulation of intestinal absorption of Ca^2+^ from the luminal contents of the intestine, primarily in the jejunum and ileum, and its transfer into the circulation [41]. Ca enters the intestine epithelial cells through the TRPV6 channel, binds to calbindin (CaBP-9k), and passes to the extracellular space and blood circulation, via an ATP-dependent Ca^2+^-ATPase (PMCA1b). The expression of these genes is regulated by 1,25(OH)_2_D_3_. In the kidney, Ca^2+^ absorption and excretion by the epithelial renal cells is mediated by the TRPV5 channel, calbindin (CaBP-28k) and PMCA1b, in a way similar to that in the intestine. These proteins are also under the control of 1,25(OH)_2_D_3_ [42]. Within the bone tissue, it seems that the response of bone cells, stimulated by 1,25(OH)_2_D_3_, is probably correlated to their maturation and differentiation stage [43]. It promotes the differentiation of the mesenchymal osteoblast precursors to mature osteoblasts via the activation of *LRP5* gene and the suppression of *RUNX2* gene. Moreover, it stimulates the production of activating agents, like RANKL (receptor activator of nuclear factor kappa-B ligand) and M-CSF (macrophage-colony stimulating factor) by mature osteoblasts, which subsequently stimulate the differentiation of osteoclast precursors and hematopoietic progenitor cells to mature osteoclasts, and their activation. Osteoclastogenesis, results in bone resorption and subsequent release of Ca and P into the extracellular fluid. This process is counter forced by the production of agents like osteoprotegerin (OPG) that binds RANKL in order to preclude its osteoclastogenetic effect. The expression of OPG gene is also under the control of 1,25(OH)_2_D_3_-VDR-RXR heterodimer, which downregulates its expression.

### 2.4. Inherited Diseases Associated with Defective Extracellular Ca Sensing Mechanism

*CaSR* gene is located in chromosome 3q13.3–21, includes 7 exons and encodes for a 1078 amino acids sequence [44]. Loss of function mutations “reset” the serum Ca^2+^ concentration upward, which leads to calcium-sensing mechanisms stimulation at higher levels of serum Ca^2+^ than in normal situations, a condition known as Familial Hypocalciuric Hypercalcemia (FHH). A similar biochemical profile is presented in patients with alterations in the downstream signalling proteins Ga_11_ and the adaptor-related protein complex 2 (AP2σ) which is involved in CaSR endocytosis [45,46]. Heterozygous activating mutations result in increased sensitivity of the receptor to extracellular Ca^2+^ concentration and induce the reverse effects (Autosomal Dominant Hypocalcaemia, ADH) [47].

#### 2.4.1. Familial Hypocalciuric Hypercalcaemia

Familial Hypocalciuric Hypercalcaemia (FHH) is a rare autosomal -mainly- dominant group of hypercalcemic disorders including 3 subtypes: FHH1, FHH2 and FHH3. Patients exhibit lifelong hypercalcemia, usually asymptomatic (~70%), with very low urinary Ca^2+^ excretion (urine Ca:Creatinine clearance ratio <0.01), slightly elevated Mg^2+^ and often normal concentrations of vitamin D metabolites [45]. PTH circulating levels are mildly elevated which may lead to misdiagnosis of primary hyperparathyroidism (PHPT), since reliable distinctions are not always possible on clinical grounds. The condition is considered to be benign, however, if untreated may lead to severe symptomatic hypercalcemia, chondrocalcinosis, or nephrocalcinosis (7%), osteomalacia (~9%), gallstones and acute pancreatitis (3.5%). However, in almost 30% of FHH patients, clinical presentation can be atypical [47,48]. It is important to identify these patients to ensure that they are not subjected to parathyroidectomy, which will not correct hypercalcemia [49]. Urine ratio of Ca to creatinine (Ca:Cr) is proposed as a simple biochemical differentiate test. However, ~20% of FHH patient are not hypocalciuric and similarly ~10% of PHPT patients may display a low Ca:Cr ratio [50,51]. Additionally, vitamin D deficiency or impairment of renal function can interfere with Ca^2+^ renal excretion [52]. Therefore, mutational analysis of FHH causative genes and functional studies of derived variants is the gold standard for a proper diagnosis [53,54].

FHH-1 (MIM 145980) is the most common type accounting for approximately 65% of FHH cases and it is caused by mutations in the *CaSR* gene. Over 300 mutations have been identified in FHH-1 patients, the majority being missense and the rest harboring frameshift, insertions/deletions and nonsense mutations [47]. Notably, the considerably abnormal transcripts derived by such mutations are not always associated with the severity of clinical phenotype. Therefore, possible mechanisms of inactivating *CaSR* mutations include: impaired biosynthesis, folding and cellular protein trafficking leading to decreased cell-surface expression [55]; conformational changes and altered G-protein coupling [11] biased signalling responses switched from intracellular Ca^2+^ pathway to equally or mainly to MAPK reduction [56].

Germline heterozygous inactivating mutations of *GNA11* gene, located on 19p13.3 chromosome, are considered the genetic cause of FHH-2 (MIM 14598) [57,58]. The gene encodes for the α-subunit of G_11_ protein, a component of CaSR transduction signalling. To date, 4 probands have been reported carrying 3 different missense substitutions (Thr54Met, Phe220Ser and Leu135Gln) and one an in-frame deletion (Ile200del). FHH-2 patients exhibit a mild clinical phenotype [57,59]. In contrast, FHH-3 (MIM 600740) represents a more severe type with higher degree of hypercalcemia and hypermagnesemia. In some patients, symptomatic hypercalcemia has been reported, complicated with decreased bone mineral density, cognitive impairment and recurrent pancreatitis. The molecular defect has been identified in *AP2S1* (19q13.3), which encodes the AP2σ-subunit [60]. To date, over 60 patients have been reported and all, except from one, carried mutations affecting the Arg15 residue of the protein [47].

The identification of *CaSR* gene as a causative gene of hypercalcemic disorders, as well as experimental and functional studies provide evidence of a CaSR-mutation dosage effect on clinical phenotype [47,61]. Despite the fact that FHH-1 is an autosomal predominately dominant disorder there is a small number of patients, the offspring of consanguineous marriages, harboring homozygous *CaSR* mutations. These individuals display higher values of serum Ca^2+^ compared to heterozygous patients while their parents were normocalcemic [47]. Functional studies revealed that these mutations had a less severe effect on the receptor compared to mutations with a dominant negative effect [62].

Neonatal severe primary hyperparathyroidism (NSPHT) (MIM 239200) is a rare di- sorder that presents at birth or within the first six months of life and is characterized by severe life-threatening hypercalcemia, hypermagnesemia, increased circulating PTH levels, massive hyperplasia of the parathyroid glands and relative hypocalciuria. Mainly homozygous or compound heterozygous inactivating mutations in *CaSR* gene have been reported while ~15% of the fifty described NSHPT probands were heterozygotes [47]. Half of the 29 NSHPT mutations are missense, located mainly in the N-terminal domain of the protein. Homozygous mutations seem to affect or interrupt CaSR signalling, impair receptor structural integrity and decreased cell-surface expression. Heterozygous mutations are located in key regions for receptor activation [8,12,63]. Serum Ca^2+^ concentrations are higher in homozygous patients compared to heterozygous, while truncated mutants seem to cause increased serum Ca levels compared to missense mutations [47]. Skeletal abnormalities including demineralization, widening of the metaphyses, osteitis fibrosa and fractures may occur. Respiratory distress, failure to thrive, lethargy and dehydration are also commonly observed. In contrast to FHH, hyperplastic parathyroid, obligatory development of permanent hypoparathyroidism and high mortality rate are typically found in NSHPT, thus, total parathyroidectomy is a routine therapy option for NSHPT, following bisphosphonates to limit serum Ca before surgery [63,64].

CaSR has also been demonstrated to be a therapeutic target. Thus, cinacalcet, a positive allosteric modulator of CaSR, has entered clinical practice to treat hyperparathyroid states. Cinacalcet enhances the receptor’s sensitivity to Ca and has been proved to be an effective treatment to some NSHPT patients [65]. Of note, cinacalcet may be used as initial NSHPT treatment due to its impact both on PTH and Ca^2+^ levels [66]. FHH patients usually require no treatment, yet cinacalcet can be efficient in some symptomatic individuals [59].

#### 2.4.2. Autosomal Dominant Hypocalcaemia

Autosomal Dominant Hypocalcaemia (ADH) is a rare familial or sporadic syndrome designated as ADH type 1 (MIM 601198) and type 2 (MIM 615361), caused by activating mutations of *CaSR,* or *GNA11,* respectively. The biochemical phenotype, except from additionally short stature in few ADH2 patients, is rather replicated for both types and opposite to FHH [58,67,68,69]. Hence, ADH is characterised by hypocalcaemia, varying from mild asymptomatic to severe in about half of ADH patients and improper low or normal PTH levels. Low concentrations of PTH, which normally induce reabsorption of Ca^2+^ from the primary filtrate, result in relative hypercalciuria, which is characterized by urinary Ca:Cr ratios that are within or above the reference range. Associated complications include renal stones, nephrocalcinosis, renal impairment and basal calcifications [68]. Symptomatic patients may present with seizures in the neonatal period or later with hypocalcemic manifestations, mainly due to neuromuscular irritability, such as paresthesia, carpopedal spasm or afebrile seizures. Associated complications include renal stones, nephrocalcinosis, renal impairment and basal ganglia calcifcations. Affected patients also exhibit hyperphosphatemia, hypomagnesemia and absolute or relative hypercalciuria [68,70]. The impact of such mutations on bone metabolism has not been clarified. ADH1 patients with different gain-of-function CaSR mutations have been reported to exhibit low, normal or even high bone mineral density (BMD) [71]. Occasionally, ADH1 patients may also develop Bartter’s syndrome type V (MIM 601198), and thus, presenting additional features, such as hypokalemia, metabolic alkalosis and hyperaldosteronemia [72].

Approximately 100 different activating *CaSR* mutations have been reported, the vast majority of which are heterozygous missense substitutions. Genetic alterations seem to locate within specific hotspot sites of the CaSR protein. In particular, ADH1-causing CaSR mutations either affect the extracellular VFT domain, promoting dimer rotation that facilitates receptor activation, [73] or alter specific transmembrane domains and the intervening third extracellular loop of the CaSR, unlocking the protein from its inactive conformation [74]. In contrast to FHH-1, CaSR mutations mentioned previously, ADH1-causing CaSR mutations switch signalling responses from MAPK reduction to intracellular Ca^2+^ pathway [75]. However, twelve CaSR protein residues have been identified to accommodate both ADH1 and FHH mutations, often representing residues which modulate receptor activation and are subject to extended conformational alterations as a response to ligand binding [76]. Pertaining to ADH2, germline activating mutations in *GNA11* seem to be very rare and mainly affecting GDP/GTP exchange or G-protein binding [77,78].

Differential diagnosis of ADH from other forms of hypoparathyroidism is of high importance as vitamin D administration may deteriorate hypercalciuria and nephrocalcinosis [68,70]. Generally, therapy of asymptomatic patients is avoided. Whereas, symptomatic disease is encountered either via thiazide diuretics [79] or recombinant human PTH. Calcilytics, negative allosteric CaSR modulators, are a promising option currently in clinical trials. [79]. Murine experimental studies have demonstrated normalization of calcium responses, increased calcium and PTH concentrations and decreased renal calcium loss [80]. This blockage of renal CaSR could also have a role in other hypoparathyroid patients.

### 2.5. Inherited Diseases Associated with Defective PTH Synthesis and Action

#### 2.5.1. Familial Isolated Hypoparathyroidism, Type 1

In some rare cases of autosomal dominant or recessive hypoparathyroidism, namely Familial Isolated Hypoparathyroidism, type 1 (FIH) (MIM 146200) patients present by heterozygous, homozygous, or compound heterozygous mutation in the *PTH* gene (11p15.3-p15.1) coding for the prepro-PTH peptide. This region is critical for the movement of the newly formed prepro-PTH through the endoplastic reticulum and consequently for the full activity of PTH [81]. Patients present hypoparathyroidism, decreased serum Ca^2+^ levels, increased serum levels of Pi and increased Ca^2+^ elimination in the urine. Serum concentrations of PTH are low or undetectable and circulating levels of 1,25(OH)_2_D_3_ are usually low or low-normal. The clinical symptoms of hypoparathyroidism are synonymous with hypocalcaemia and may vary from quite mild to more severe muscle cramps and convulsions. FIH is also a feature common to a variety of hereditable syndromes, such as ADH type 1 and 2 (described above), or Familial Isolated Hypoparathyroidism-2 (MIM 618883) due to homozygous mutations in “glial cells missing transcription factor-2” gene (*GCM2*) [82,83]. However, it should be noted that the most common cause of hypoparathyroidism is the loss of parathyroid tissue after surgical removal of the parathyroid glands. Autoimmunity, isolated or associated with other endocrine diseases (autoimmune polyendocrinopathy candidiasis ectodermal dystrophy, APECED) and developmental disorders of the parathyroid glands (i.e., DiGeorge syndrome, Charge syndrome, Hypoparathyroidism deafness and renal dysplasia (HDR), Kenny-Caffey syndrome, Kearns-Sayre) may also be causes of hypoparathyroidism [84].

#### 2.5.2. Jansen Metaphyseal Chondrodysplasia

Activating mutations in residues His223 and Thr410 of the PTHR1 (3p21.31) have been associated with Jansen metaphyseal chondrodysplasia (#MIM156400). It is a rare autosomal form of short-limbed dwarfism, in which neonates appear normal but have radiographic and laboratory abnormalities [85]. They present with metaphyseal chondrodysplasia, hypercalcaemia, hypophosphatemia, undetectable serum levels of PTH and PTHrP, decreased renal tubular reabsorption of P and increased cAMP in urine. Other laboratory findings reveal high level of 1,25(OH)_2_D_3_, ALP level and urine hydroxyproline. PTHR1 ligand analogues, such as DTrp12-PTH(7–34), function as inverse agonists, which can inhibit constitutively active receptors, and thus, could prevent homeostatic abnormalities occurring in these patients [20].

#### 2.5.3. Pseudohypoparathyroidism

PTH, as well as other peptide hormones i.e., thyroid stimulating hormone (TSH), use the α subunit of stimulatory guanine-nucleotide binding protein encoded by *GNAS* gene (20q13.2–13.3) to enhance cAMP production via adenylyl cyclase signalling [86]. Heterozygous mutations in *GNAS* gene lead to the production of inactive Gαs or to small amounts of active Gαs, while homozygous deficiency of Gαs is incompatible with life. In this group of disorders, PTH is correctly synthesized, secreted and bound to a well-functioned receptor. However, its binding does not transduce any signal downstream, a condition known as pseudohypoparathyroidism (PHP) [87].

PTH resistance-basically on the proximal renal tubules- is the hallmark of PHP. Patients with PHP present with hypocalcaemia, hyperphosphatemia, diminished serum concentration of 1,25(OH)_2_D_3_, and increased serum PTH concentration [87,88]. Today, several PHP variants have been identified: PHP type I (1a, 1b, 1c), pseudopseudohypoparathyroidism (PPHP) and PHP type II [88]. In PHP-I types, administration of exogenous biologically active PTH fails to generate, either a hyperphosphaturic or increased cAMP excretion. Whereas, in the PHP-II type, patients have normal cAMP and impaired phospaturic excretion.

All PHP-Ia (MIM 103580) patients possess heterozygous mutations for *GNAS* gene resulting in diminished or no cAMP generation. In these patients, there is a markedly attenuated urinary cAMP response (~50%) to exogenous administration of PTH. Furthermore, patients with PHP-Ia present with developmental and somatic defects as a whole are referred to as Albright’s Hereditary Osteodystrophy (AHO) [88,89]. Short stature, round face, brachydactyly, obesity, subcutaneous calcifications and cognitive abnormalities are classic features of AHO. Albright and colleagues, noticed that some patients with the above combination of symptoms had normal Ca, P and PTH serum levels. This variant phenotype is termed PPHP and results from the same mutations in *GNAS,* which lead to AHO phenotype without PTH resistance [89]. The variation of the aspects of the phenotype depends on the parental origin of the mutation reflecting its imprinted expression [90]. Patients who inherit the defective gene from the father have PPHP (MIM 612463), whereas those who inherit the same mutation from the mother have PHP Ia. For instance, in renal proximal tubules, Gαs expression from the paternal allele is silenced, and transcription takes place from the maternal allele. When an inactivating mutation is inherited from a female carrier, the level of Gas in this tissue are affected and hormone resistance occurs (PHPIa). If the defective allele is transmitted from the male carrier, the expression of Gαs remains unaltered. However, other dysfunctions of those patients occur after maternal and paternal transmission, indicating haploinsufficiency of Gαs in some tissues in which Gαs expression is biallelic. AHO phenotype is also observed in PHP1c type (MIM 612462). Patients classified as PHP1c cases present the same features as PHP1a except the retained erythrocyte Gαs activity [91].

Patients with PHP type 1b (MIM 603233) lack AHO features and manifest isolated renal resistance to PTH and sometimes mild TSH resistance. Most cases are sporadic, but some cases (10–15%) are familial and show an autosomal dominant mode of inheritance. In familial cases, PTH resistance develops after maternal transmission of the genetic defect, as is the case of PHPIa. Most patients with PHPIb show abnormal patterns of methylation in the differentially methylated regions (DMRs) associated with the *GNAS* complex locus (20q13) [92,93,94]. *GNAS* shows differential methylation at four distinct DMRs: one paternally methylated-DMR (*GNAS-NESP*: TSS-DMR) and three maternally methylated-DMRs (*GNAS-AS1*:TSS-DMR, *GNAS-XL*:Ex1-DMR and (*GNAS A/B*:TSS-DMR) [87]. Familial cases, present usually loss of methylation at *GNAS A/B*:TSS-DMR, secondary to a 3 kb microdeletion on the maternal allele of *cis*-acting control elements within *STX16* [94]. In nearly all sporadic cases of PHP1B, two or more DMRs, in addition to *GNAS A/B*:TSS-DMR, are also affected [93].The severity of PHP type 1b can vary considerably from one patient to another even within single kindred. Members of the affected family who share the same haplotype have been reported to be clinically asymptomatic with normal Ca levels.

PHP type II is a clinically heterogenous syndrome. Only a few cases have been reported and the molecular defect remains elusive. Patients with PHP type II present with hypocalcaemia, decreased serum 1,25(OH)_2_D_3_, reduced or absent phosphaturic response to exogenous PTH, and a normal increase in urinary cAMP excretion [95].

### 2.6. Inherited Disorders Associated with Defective Vitamin D Metabolism

#### 2.6.1. Vitamin D Hydroxylation Deficient Rickets, Type 1A, or Pseudovitamin D Deficiency Rickets, or 1α-Hydroxylase Deficiency

Vitamin D Hydroxylation Deficient Rickets, Type 1A (VDDR1A) (MIM 264700) is a rare autosomal recessive disease caused by mutations in *CYP27B1* which result in diminished or complete absence of 1α-hydroxylase activity [96]. The biochemical findings are hypocalcaemia, hypophosphatemia, elevated PTH levels due to secondary hyperparathyroidism, increased ALP activity and low urinary Ca^2+^ excretion. Patients have normal or elevated 25(OH)D levels, but low or undetectable levels of 1,25(OH)_2_D_3._ The disorder usually manifests in early life with growth retardation, bone and joint deformities, teeth hypoplasia, hypotonia and muscle weakness. Severe hypocalcaemia can result in seizures and tetany. X-rays reveal classical features of rickets. The low serum levels of 1,25(OH)_2_D_3_ and the therapeutic response to physiological doses of exogenous 1,25(OH)_2_D_3_ distinguish 1α-hydroxylase deficiency from Vitamin D-dependent rickets type 2A discussed below [97].

#### 2.6.2. Vitamin D-Dependent Rickets Type 2A

Vitamin D-Dependent Rickets Type 2A (VDDR2A) (MIM 277440) is a rare, clinical entity caused by loss-of-function mutations in the VDR gene, resulting in generalized resistance of target tissues to 1,25(OH)_2_D_3_ [37,96,97,98]. The human VDR is the product of a single chromosomal gene located on chromosome 12 at 12q13–14. It contains 10 exons that code for DNA binding (DBD), hormone binding (LBD) and heterodimerization domains. Mutations in the DBD are associated with complete hormone unresponsiveness whereas the mutations found in the LBD results in heterogeneous degree of resistance to 1,25(OH)_2_D_3_ [37]. The disorder follows an autosomal recessive pattern of inheritance and presents with severe rickets, growth retardation, teeth hypoplasia, typical bone deformities like bowed legs and rachitic rosary and occasionally, alopecia. Alopecia can be present at birth or can develop later, during the first year of life. The exact pathophysiologic mechanism causing alopecia is not well-understood. Numerous studies have shown that alopecia is not correlated with Ca^2+^ or Vitamin D levels, but is associated with the dysfunctional receptor, supporting the role of the receptor in several metabolic pathways. It has been proposed that mutations, affecting DNA binding or RXR heterodimerization cause alopecia, while mutations that prevent the binding of 1,25(OH)_2_D_3_ or coactivators to the receptor are not associated with alopecia [98,99,100]. The biochemical findings include hypocalcaemia, hypophosphatemia, secondary hyperparathyroidism and elevated elevated levels of serum 1,25(OH)_2_D_3_ and ALP.

Intravenous calcium administration, followed by high doses of oral calcium is a successful method of treatment of VDDR2A [98]. In some cases, 1,25(OH)_2_D_3_ and vitamin D analogues are used [97]. Recently, cinacalcet has been demonstrated to be effective in normalizing hypophosphatemia and the hyperparathyroidism and ameliorate the bone pathology in some patients [101,102]. Spontaneous healing during adolescence has also been described [103]. The desirable laboratory findings after successful treatment are restoration of Ca^2+^ and Pi to normal levels as well as the decrease in PTH and ALP levels. P levels usually normalize after treatment with Ca, suggesting that hypophosphatemia is mostly associated with the secondary hyperparathyroidism.

## 3. Phosphorus and Its Partners

Phosphorus (P) is an essential mineral for many biological processes including bone development and metabolism, cell signaling, and muscle contraction. It is also indispensable for cellular structures, nucleic acids, lipids and proteins. Daily P uptake varies between 800–1500 mg, depending of diet. About 65% of this quantity is absorbed in duodenum and jejunum via a paracellular and an active transcellular process through the sodium phosphate cotransporter 2b (NaPi-2b), which is controlled by 1,25(OH)_2_D_3_ and dietary P itself [104]. The skeleton is the main site of P storage in the human body (80–85% of total P), while approximately 15% of the mineral is distributed in extraskeletal sites. In extracellular fluid (1% of total P), it is in the form of inorganic phosphate (Pi). The normal serum Pi concentration ranges from 3.4 to 4.5 mg/dL (1.12 to 1.45 mmol/L). In children, varies from 4 to 7 mg/dL with higher values in neonatal period due to renal immaturity. Renal function is fundamental to Pi serum handling as most of it, is filtered at the glomerulus (about 7gr daily); 80–90% is reabsorbed by renal tubules (75% in proximal and 10% in distal tubule) and the remainder (~700 mg) is lost in urine, a quantity equal to intestinal absorption. The reabsorption takes place via two transporters on the apical membrane of the proximal tubule, NaPi-2a and NaPi-2c [105,106] regulated by PTH and FGF23 [107].

Although Ca homeostasis has been extensively elucidated, Pi homeostasis is less well understood. PTH, 1,25(OH)_2_D_3_, and FGF23 are the main regulators of bone, renal and intestinal Pi handling. Recently, it has been shown that hyperphosphatemia inhibits the CaSR in a noncompetitive manner and thus stimulates PTH secretion [108] which in turn increases renal excretion of Pi. Circulating PTH acts through its receptor PTHR1 at the target organs, as described previously for Ca^2+^ homeostasis. Moreover, PTH causes a differential decrease in the abundance of the cotransporters NaPi-2a and NaPi-2c, via a PTH-induced internalization with the synergy of a myosin motor [107]. All molecular mechanisms associated with PTH end by decreasing serum Pi concentration. On the other hand, hypophosphatemia and low serum PTH levels induce 1-α-hydroxylation in the kidney, increasing 1,25(OH)_2_D_3_ synthesis, which subsequently increases Pi intestinal absorption [109] and renal Pi reabsorption, as described previously.

FGF23 seems to hold a central role in the bone-kidney axis [110]. It is synthesized mainly by osteocytes and to a lesser extent by osteoblasts and acts mainly in kidneys and parathyroid glands as a phosphatiuric factor, inhibitor of 1,25(OH)_2_D_3_ and modulator of PTH synthesis and secretion [111]. 1,25(OH)_2_D_3_ and extracellular Ρi levels enhance *FGF23* transcription [112,113,114]. In the context of a negative feedback loop, FGF23 downregulates 1,25(OH)_2_D_3_ by both inhibiting 1α-hydroxylase and stimulating 25-hydroxyvitamin D-24-hydroxylase in the kidney. Moreover, FGF23 suppresses the expression of NaPi-2a and NaPi-2c cotransporters on the apical membrane of proximal tubules. As a result, it decreases renal Pi reabsorption [115,116]. FGF23 excess leads to several hypophosphatemic diseases featured by renal Pi wasting and rickets/osteomalacia, while deficient actions of FGF23 result in hyperphosphatemic tumoral calcinosis with enhanced renal Pi reabsorption.

FGF23 actions in the tissues are mediated by FGF receptors (FGFRs), mainly types 1, 3 and 4. Notably, some studies have demonstrated that FGFRs are also significant for FGF23 synthesis [117,118]. FGFR1 is the main receptor via which the FGF23 exerts its renal phosphaturic effect [115]. Following the observation that FGFRs binds with low affinity to FGF23 [119], in vivo and in vitro studies revealed a single pass transmembrane protein, named αKlotho, as an obligate co-receptor. αKlotho has been demonstrated to form a complex, mainly with FGFR1, enhancing receptor’s FGF23-mediated activation [120,121,122]. Experimental studies on genetic modified mice suggest a collaboration between FGF23 and αKlotho to modulate mineral homeostasis, mostly via their renal effect [120,123]. Therefore, in the kidneys, Klotho mediates the hypophosphatemic action of FGF23 and the downregulation of calcitriol, while in the parathyroid glands it modulates PTH expression [124].

### 3.1. Hypophosphatemic Rickets with Normal or Decreased FGF23 Levels

Hereditary Hypophosphatemic Rickets with Hypercalciuria

Hereditary Hypophosphatemic Rickets with Hypercalciuria (HHRH) (MIM 241530) is an autosomal recessive disorder with a likely underestimated incidence of 1:250,000, firstly described in a consanguineous kindred [125,126]. Later, inactivating, either homozygous or compound heterozygous, mutations in the *SLC34A3* gene have been identified as the genetic cause of the disorder [127,128]. *SLC34A3* is located in chromosome 9q34.3 which encodes the renal sodium-phosphate cotransporter (NaPi-2c). Approximately 40 mutations affecting more than 35 kindreds have been described [129,130]. Deletions (228delC, 905delC) predicting a prematurely terminated translation product and missense mutations (R353L, A413E, G196R, R468W), are most commonly reported [127,128,131].

Generally, impaired function of Na-P cotransporter results in P wasting and hypophosphatemic rickets, short stature, bowing, normal or reduced FGF23 levels, hypercalciuria, elevated 1,25(OH)_2_D_3_ and low PTH levels [129,132]. Elevated 1,25(OH)_2_D_3_ promotes intestinal Ca absorption leading to hypercalcaemia [129,133] and helps distinguish this disorder from other types of phosphaturic rickets. Furthermore, decreased PTH-dependent Ca-reabsorption in the distal renal tubules contributes to increased renal Ca^2+^, which along with Pi excretion, results in the development of nephrolithiasis in almost half of HHRH patients. Treatment consists of P administration in order to improve bone mineralization and reduce 1,25(OH)_2_D_3_ levels. Therapy with vitamin D analogues is contradicted, as it enhances hypercalcemia and hypercalciuria [129].

### 3.2. Hypophosphatemic Rickets with Elevated FGF23 Levels

#### 3.2.1. X-Linked Hypophosphatemic Rickets (XLH)

XLH rickets (*MIM 307800)* is inherited with X-linked dominant pattern and consists the most common form of hereditary rickets with an incidence of 1:20,000 [134]. Loss-of-function mutations in *PHEX* gene have been identified as its causative genetic defect. *PHEX* (Phosphate regulating with Homologies to Endopeptidases on the X-chromosome), gene is located in chromosome Xp22.1 and is highly expressed in skeleton [135,136]. More than 300 distinct pathogenic variations have been reported [116], leading to FGF23 excess, hypophosphatemia and phosphaturia and inappropriately low levels of 1,25(OH)_2_D_3_.

Patients usually exhibit rickets, short stature, growth retardation, bowing of the lower extremities and during adulthood osteomalacia, dental abscesses ectopic calcification, mineralizing enthesopathy and osteoarthropathy [116,135]. Conventional therapy includes multiple doses of oral P supplementation and active vitamin D (calcitriol, alfacalcidol, paricalcitol, eldecalcitol), in order to increase intestinal Pi reabsorption. This therapy can be complicated by hypercalciuria, nephrocalcinosis and secondary hyperparathyroidism. Thiazide diuretics have been used to reduce the risk of nephrocalcinosis, while the calcimimetic drug cinacalcet has been suggested in patients who have developed secondary or tertiary hyperparathyroidism [137,138]. In 2018, burosumab (Crysvita^®^; Kyowa Hakko Kirin Co., Ltd. and Ultragenyx Pharmaceutical Inc., Novato, CA, USA), a human neutralizing antibody for FGF23 was approved for the treatment of XLH in adults and children older than 1 year and in 2020 for the treatment of tumor induced osteomalacia [139,140]. Burosumab outweighs conventional therapy in children with XLH pertaining to rickets severity, growth and biochemistries [141].

#### 3.2.2. Autosomal Dominant Hypophosphatemic Rickets

Autosomal Dominant Hypophosphatemic Rickets (ADHR) (MIM 193100) is a rare disorder that follows autosomal dominant inheritance with incomplete penetrance. FGF23 gene is located in chromosome 12p13.3, encompassing 3 exons and the derived protein consists of 251 amino acids including a signal peptide of 24 aa in the N-terminal. Under physiological circumstances, FGF23 is present in serum in the active form of an intact peptide (25th to 251th aa). FGF inactive fragments are derived after proteolytic processing by endopeptidases that recognize a specific subtilisin-like proprotein convertase (SPC) cleavage site R^176^XXR^179^ [142]. Activating mutations in this site are the causative genetic defect of ADHR, while inactivating mutations result in hyperphosphatemia and elevated 1,25(OH)_2_D_3_ levels [143]. Increased FGF23 activity results in suppressed co-transporters NaPi-2a and NaPi-2c, as well as in decreased 1a hydroxylase expression and hypophosphatemia [143].

Clinical manifestations and biochemical findings resemble XLH. However, affected patients may present with a variable phenotype. Patients with a childhood onset may develop severe rickets accompanied by short stature and bone deformities. While others may appear with delayed onset of the disease in adolescence or adulthood and milder symptoms, like muscle weakness and bone pain, without limb deformities [144,145]. FGF23 levels have been reported to correlate with the disease’s severity. Spontaneous normalization of serum P levels has also been reported [146]. Converging data suggest that the clinical phenotype of ADHR is temporally associated to the existence of iron deficiency, as iron status seems to influence FGF23 concentrations [144,147]. ADHR therapeutic options are similar to XLH conventional therapy along with recognition and treatment of iron deficit [148]. Although burosumab is a promising regimen for the remainder of FGF23-mediated disorders, its efficiency has to be proved in clinical trials [149].

#### 3.2.3. Diseases Associated with *FGFR1* Gene Mutations

Osteoglophonic Dysplasia (MIM166250), a very rare type of dwarfism, is caused by gain of function mutations of *FGFR1.* Hypophosphatemia and low 1,25(OH)_2_D_3_ levels accompanied by chondrodysplasia, craniosynostosis and spondyloepimetaphyseal dysplasia in imaging studies are disease’s characteristic manifestations [150]. Mutations in this receptor have been also associated with a spectrum of phenotypically different rare syndromes such as Pfeiffer, Kallmann, Trigonocephaly, Jackson–Weiss and Hartsfield [151].

#### 3.2.4. Autosomal Recessive Hypophosphatemic Rickets

Autosomal Recessive Hypophosphatemic Rickets (ARHR) type 1 (MIM 241520) and 2 (MIM 613312) are associated with molecular defects in Dentin matrix protein 1 (DMP1) and the ectonucleotide pyrophosphatase phosphodiesterase 1 (ENPP1), respectively [152,153]. *DMP1* is located in chromosome 4q21.1 and 13 distinct mutations have been reported in 32 patients of 16 families. DMP1 belongs to the SIBLING (Small Integrin Binding Ligand N-linked Glycoprotein) protein family, a group of non-collagenous extracellular matrix proteins and is expressed in osteoblasts and osteocytes, promoting proper growth and formation of hydroxyapatite crystals [154]. DMP1 is cleaved into two fragments of 35 and 57 kDa. The C-terminal fragment of 57 kDa appears to suppress FGF23 expression [155], while patients harboring mutations that affect the *DMP1* fragment present with shorter stature and lower serum Pi level, compared to those harboring mutations only affecting N-terminal fragment of the protein [156]. Overall, clinical findings of ARHR patients are similar to those appearing in other types of hypophosphatemic rickets. Interestingly, there are some hypophospatemic patients, carriers of a *DMP1* mutation, who present with increased bone density and overgrowth of tubular bones [156].

*ENPP1*, located in chromosome 6q22.23, encodes for a plasma protein which controls the generation of inorganic pyrophosphate (PPi), which inhibit hydroxyapatite crystal deposition by hydrolyzation of ATP to AMP [157]. *ENPP1* has also been identified as the causative gene of another entity, generalized arterial calcification in infancy (GACI1), presenting precociously with arterial calcification [158]. However, patients with ARHR do not develop GACI, probably due to low P levels [153]. Clinical features resemble other types of hypophosphatemic rickets, while primary hyperparathyroidism has been also described in a recent report [159].

Mutations in *FAMC20* gene have been recently associated with a nonlethal variant of Raine syndrome as a novel form of FGF23-related hypophosphatemia and hyperphosphaturia and proposed to represent a third form of ARHR [160,161]. FAMC20 is a Golgi kinase supported to be involved in the post-translational modification of FGF23. FAMC20 phosphorylates FGF23 and promotes its cleavage, while a recent report suggests the SIBLING-proteins to be substrates for FAM20C. Clinically additional finding include severe dental anomalies, osteosclerosis, intracerebral calcifications and dysmorphic features [160].

Treatment of ARHR is similar to the conventional treatment of the other hypophosphatemic disorders already discussed above.

#### 3.2.5. Diseases Associated with Klotho Gene Mutations

The genetic evaluation of a patient with hypophosphatemic rickets and increased plasma soluble Klotho, revealed a translocation between chromosome 9 and 13, with a breakpoint in vicinity to Klotho (*KL*) gene. Besides elevated Klotho levels, patients displayed increased enzymatic activity protein and FGF23 concentrations accompanied by clinical and biochemical findings of hypophosphatemic rickets [162]. Interestingly, the patient presented with parathyroid hyperplasia and markedly elevated PTH levels. The underlying mechanism of this manifestation remains elusive.

On the other hand, Familiar Tumor Calcinosis (FTC), a rare autosomal recessive disorder, is a mirror image of FGF23 related hypophosphatemic diseases, featured by hyperphosphatemia, inappropriately elevated 1,25(OH)_2_D_3_and PTH levels and ectopic calcified masses, [163]. The genetic defect of the disorder is loss-of-function mutations either in *KL* gene or FGF23 or N-acetyl-galactosaminyltransferase (GALNT3)—an enzyme involving in FGF23 O-glycosylation. In *KL* gene, only one a missense mutation has been reported in a single FTC patient [131].

## 4. Conclusions

Inherited disorders affecting Calcium and Phosphorus metabolism are mainly rare diseases, often with overlapping clinical and biochemical features, thus, making their diagnosis challenging. In this review, we aim to connect the known molecular alterations affecting the actors of Ca/P signalling, their effects on the molecules downstream, and their impact on the biochemical profile of the patients. The knowledge of the genetic basis helps in understanding the biology and the aetiopathogenesis of the diseases and therefore ensures more accurate diagnosis and therapeutic approach. The study of the CaSR and the Ca sensing mechanisms resulted in new therapeutic options—calcimimetics or calcilytics that modulate the activity of the receptor. The identification of the role of FGF23 and PHEX improved the conventional therapy for XLH, since a human neutralizing antibody for FGF23, burosumab, was approved for the treatment of XLH and tumor induced osteomalacia. It seems that there are still unknown interactions in this complex network between bone-kidney-intestine and parathyroid glands that are waiting to be discovered.
